# Current trends in mouse models of glioblastoma

**DOI:** 10.1007/s11060-017-2626-2

**Published:** 2017-10-20

**Authors:** Masafumi Miyai, Hiroyuki Tomita, Akio Soeda, Hirohito Yano, Toru Iwama, Akira Hara

**Affiliations:** 1 Department of Tumor Pathology, Gifu University Graduate School of Medicine, 1-1 Yanagido, Gifu, 501-1194 Japan; 2 Department of Neurosurgery, Gifu University Graduate School of Medicine, 1-1 Yanagido, Gifu, 501-1194 Japan

**Keywords:** Glioblastoma, Mouse, Xenograft, Glioma

## Abstract

Glioblastoma is the most deadly brain tumor type and is characterized by a severe and high rate of angiogenesis, remaining an incurable disease in the majority of cases. Mechanistic understanding of glioblastoma initiation and progression is complicated by the complexity of genetic and/or environmental initiating events and lack of clarity regarding the cell or tissue of origin. To determine these mechanisms, mouse models that recapitulate the molecular and histological characteristics of glioblastoma are required. Unlike in other malignancies, viral-mediated mouse models of glioblastoma rather than chemically induced mouse models have been developed because of its sensitivity to viruses. Based on recent molecular analyses reported for human glioblastoma, this review critically evaluates genetically engineered, xenograft, allograft, viral-mediated, and chemically induced mouse models of glioblastoma. Further, we focus on the clinical value of these models by examining their contributions to studies of glioblastoma prevention, tumorigenesis, and chemoresistance.

## Introduction

Glioblastoma is the most common and deadly primary brain tumor [1], and the most aggressive type of glioma, a collection of tumors arising from glial cells. It is also termed glioblastoma multiforme because of its complex phenotype. The current standard of care is based on maximal safe surgical resection and concurrent chemoradiation with temozolomide, followed by maintenance chemotherapy, resulting in median survival rates of approximately 15 months [2].

Over the past few years, genomic and proteomic characterization along with robust animal models of glioblastoma have provided invaluable data. In addition, pre-clinical models and a better understanding of the core pathways disrupted in glioblastoma are providing renewed optimism for novel strategies targeting these devastating tumors. Here we discuss the current advances in rodent models, particularly mouse models, of glioblastoma and other gliomas, and how these developments have influenced strategies for therapeutic intervention.

## Pathological features of glioblastoma

The 2016 World Health Organization Classification of Tumors of the Central Nervous System uses molecular parameters in addition to histology to define many tumor entities [3]. In this new classification, diffuse gliomas include WHO grade II and grade III astrocytic tumors, grade II and III oligodendrogliomas, and grade IV glioblastomas.

Glioblastomas are divided into three categories in the 2016 CNS WHO classification according to a key genetic prognostic marker, isocitrate dehydrogenase (IDH): glioblastoma, IDH-wildtype; glioblastoma, IDH-mutant; glioblastoma, NOS. IDH-wildtype (about 90% of cases) corresponds most frequently with clinically defined primary or de novo glioblastoma and predominates in patients over 55 years of age [4]. IDH-mutant glioblastoma (about 10% of cases) corresponds closely to so-called secondary glioblastoma with a history of lower-grade diffuse glioma, and preferentially arises in younger patients [4]. Glioblastoma NOS is a diagnosis that is reserved for tumors for which full IDH evaluation cannot be performed or for which test results remain inconclusive.

Pathological characteristics of glioblastoma is typically a highly cellular glioma, usually comprised of poorly differentiated, sometimes pleomorphic tumor cells with nuclear atypia and vigorous mitotic activity. Conspicuous microvascular proliferation and/or necrosis is an essential diagnosis characterization.

Molecular characteristics typical of IDH-wild type glioblastoma include TERT promorter mutations (present in ~ 80% of cases), homozygous deletion of CDKN2A/CDKN2B (~ 60%), loss of chromosomes 10p (~ 50%) and 10q (~ 70%), EGFR alterations (i.e. mutation, rearrangement, altered splicing, and/or amplification; ~ 50%), PTEN mutations(25–30%), and PI3K mutations (~ 25%) [4, 5].

## Mouse models of glioblastoma

The use of mice to create suitable models for the study of specific tumors or to investigate the role of candidate genes has obvious advantages. Firstly, manipulation of the mouse genome to create specific genetic changes by microinjection of DNA into fertilized eggs or by homologous recombination in embryonic stem cells is relatively easy compared with other mammalian species such as rats. Another advantage is the availability of inbred strains of mice that are genetically identical, obtained by breeding sibling mice over 20 generations. Since these animals present the same genetic background, they can be compared for their response to a treatment or a genetic modification between different laboratories. The laboratory mouse shares extensive molecular and physiological similarities to humans and is a powerful tool for studying cancer.

Transgenic mouse models offer an opportunity to develop and utilize an easily replenished, reproducible, spontaneously manipulated, and more accurate pre-clinical model of human cancers, which we can use to enhance our molecular knowledge and to test promising therapies. Therefore, mouse models that recapitulate human glioblastoma may be an invaluable tool. However, these conventional genetic approaches, such as transgenics and knockouts, are limited by the time and costs associated with extensive intercrossing of mouse lines. Several new viral vector-mediated genetic approaches offer the ability to directly modify the genome of somatic cells in mouse tissues and these have recently been applied to the rapid generation of complex mouse tumor models that harbor multiple genetic changes. On the other hand, xenograft and allograft models can be used to measure therapeutic responses to drugs more rapidly than genetically engineered or viral vector-mediated models. Chemical carcinogen-induced models are usually generated in rats, and only a small number of instances of chemical carcinogen-induced models are currently known.

### Genetically engineered models

The molecular progression of gliomas, like many tumors, involves the accumulation of genetic and epigenetic alterations that result in the loss of tumor suppressor gene function (*PTEN, TP53, CDKN2A, RB*) or the activation of oncogenic pathways (p21–RAS, PI3K, EGFR, CDK4, MDM2) [6–8].

There are several examples of aberrant expression of relevant downstream signaling pathways in mouse glioma modeling. These include astrocytomas of varying grades resulting from glial fibrillary acidic protein (GFAP)-regulated expression of v-src [9]. Weissenberger et al. [9] generated a transgenic mouse model for low-grade astrocytoma (early) and high-grade astrocytoma (later) by expressing v-src kinase under the control of *GFAP* gene regulatory elements in astrocytes.

Ding et al. [10, 11] utilized our initial observation of aberrant activation of the p21–RAS signaling pathway in astrocytomas to develop glioma models using ES transgenesis [11]. Since wild-type EGFR and mutant EGFRvIII are the most common gain-of-function alterations in malignant human astrocytomas, they reported the generation of mice expressing these proteins under the regulation of the *GFAP* promoter [10, 11].

When the glioblastoma-like tumors are examined in these mice, additional genetic alterations such as those found in human glioblastomas (overexpression of *EGFR, CDK4, MDM2*; decreased expression of *CDKN2A, TP53, PTEN*) are present [12].

Zhu et al. [13] reported that loss of *TP53* and activation of the RAS pathway via *NF1* inactivation in CNS cells is sufficient to cause malignant astrocytoma formation with 100% penetrance.

Overexpression of relevant oncogenic receptors or downstream signaling pathways has also been employed in the development of mouse glioma models. These have been either alone or in combination with mice harboring specific knockouts of relevant cell cycle regulatory proteins. For example, S100 glial precursor promoter-regulated v-ERBB (an activated member of the EGFR family) transgenic mice develop oligodendrogliomas, which are potentiated in terms of shorter latency and increased malignancy when initiated in mice deficient for both p16 and p19 (*Cdkn2a*-null mice) [14].

Briefly, Bardella et al. [15] have established small nodules like glioma in the subventricular zone. Nes-CreER^(T2)^; Idh1fl^(R132H)/+^ developed small nodules (up to 1 mm diameter) originating from the subventricular zone at 2–6 weeks after tamoxifen injection.

The nodules expressed proliferation markers, such as Ki67, and retained BrdU label, suggesting that they exhibited a variety of proliferative behaviors. Further, many nodule cells also expressed the astrocytic and NSC marker GFAP and in some lesions, a few cells expressed the neuroblast marker Doublecortin (Dcx).

Table 1 summarizes the currently used and relevant glioma mouse models that recapitulate the hallmarks of human glioblastoma.

**Table 1:** Genetically engineered and viral vector-mediated transduction mouse models of human glioma

Tumor classification	Transgene	Knockout, knockin	Grade	Incidences	Study
Small nodule like glioma	NES-CreER^(T2)^	IDH1 ^R132H^ knockin	NA*	100% by 2–6 weeks	Bardella et al. [15]
Low-grade astrocytoma	*Src* transgene		II	14% by 2.5–65 weeks	Weissenberger et al. [9]
	*GFAP*-*HRAS* ^V12^		II	95% by 16–24 weeks	Ding et al. [10]
High-grade astrocytoma	*Src* transgene		III	10–20% at later	Weissenberger et al. [9]
	*GFAP*-*HRAS* ^V12^	Floxed *NF1* + *Trp53* knockout	III–IV	100% by 2–16 weeks	Ding et al. [10]
	*GFAP*-T121 transgene	*PTEN* ^+/−^	II–III	100% by 4–32 weeks	Xiao et al. [55]
	*GFAP*-Cre	*NF*1 + *Trp53 cis*	II–IV	30–75% by 15–55 weeks	Reilley et al. [56]
	*HRAS* ^V12^ and *AKT*		III–IV	40% by 16–20 weeks	Marumoto et al. [23]
Glioblastoma	*GFAP*-Cre	Floxed *NF1* + *Trp53* knockout	II–IV	100% by 10–45 weeks	Zhu et al. [13]
	*Kras* and *AKT* (RCAS virus)	*Cdkn2a* knockout	IV	42–49% by 12 weeks	Uhrbom et al. [18]
	*PDGFB* (RCAS virus)	*Cdkn2a* knockout, *Trp53* knockout	IV	100% by 4–7 weeks	Hambardzumyan et al. [19]
	*EGFR*vIII(Ad-Cre virus)	*PTEN* ^*F*/*F*^	II–IV	93% by 6–15 weeks	Wei et al. [21]
	*HRAS* ^V12^ and *AKT*	*Trp53* knockout	IV	100% by 10–13 weeks	Marumoto et al. [23]
	*NES*-CreER	Floxed *NF1*, Floxed *PTEN*, Floxed *Trp53*	III–IV	100% by 24–56 weeks	Alcantara Llaguno et al. [24]
	*EGFR*vIII(Ad-Cre virus)	*Cdkn2a, PTEN* ^*F*/*F*^	IV	100% by 5–13 weeks	Zhu et al. [57]
Low-grade oligodendroglioma	S100b-vERBB transgene		II	75% by 52 weeks	Weiss et al. [14]
	*PDGFB* (RCAS virus)		II	60% by 12 weeks	Dai et al. [20]
High-grade oligodendroglioma	S100b-vERBB transgene	*Cdkn2a* knockout	III	90% by 4–24 weeks	Weiss et al. [14]
	*GFAP*-*HRAS* ^V12^ + *GFAP*-*EGFR*vIII		III	100% by 2–13 weeks	Ding et al. [11]
Diffuse intrinsic pontine glioma	*PDGFB* + H3.3K27M (RCAS virus) Pax3-Tv-a	*Trp53* knockout	II–IV	73% by 5–12 weeks	Misuraca et al. [25]

*not applicable

### Viral vector-mediated transduction model

In recent years, viral vectors have been extensively used for the generation of mouse models of interest in the study of brain tumors. Several routes for viral vector delivery to the brain are available: intracerebral stereotaxic injection, intrathecal and intraventricular injection, and intravascular infusion with or without modification of the blood–brain barrier. The choice of route for viral vector administration needs to be carefully considered since it affects neuronal cell transduction efficiency and spatial distribution, as well as the level of transgene expression in the infected cells [16]. Intracerebral injection offers the advantages of low toxicity, high local vector concentrations, and localized transgene delivery, but it does not allow wide viral vector distribution and requires invasive surgical intervention. Ubiquitous distribution of viral vectors in the CNS could be achieved by intrathecal or intraventricular injection but these methods do not permit spatial selectivity of delivery and require a large amount of vectors. Finally, intravascular viral vector applications do not require invasive surgical intervention but necessitate the use of high vector concentrations because of losses in peripheral organs such as the liver.

Virally transduced expression of relevant gain-of-function alterations, in combination with transgenic mouse technology, allows one to model such somatic alterations at a later stage in life, though it does not lead to germline colonies. Although the link between a viral etiology and human gliomas is weak, retroviruses that have been engineered to express relevant gain-of-function genes have been used to create glioma models in mice and other mammals [17, 18]. This includes members of the Rous sarcoma virus family and simian sarcoma virus, whose transforming properties result from overexpression of the viral oncogene v-sis, the cellular counterpart of which is c-sis or PDGF-B.

Retroviruses carrying v-sis (*PDGFB*) injected into normal mice have yielded astrocytic tumors, with varying glioma types generated when injected in *Cdkn2a*-null mice. One of the best examples of coupling retroviruses to express somatically defined gain-of-function genes in varying cell lineages and genetic backgrounds to model gliomas is the RCAS-tva system [19]. This system results in focal gliomas, the subtype and grade of which varies with the injected retrovirally transduced gene (i.e. *PDGFB, EGFR*vIII, activated p21–*RAS*, activated *AKT*), the lineage of the cell expressing the tva receptor (*GFAP, NES*) and underlying genetic cell cycle alterations in the mice (null for *Cdkn2a, Trp53* etc.). For example, retrovirally transduced expression of v-sis or *PDGFB* in *GFAP*-tva mice resulted in oligodendrogliomas or mixed oligoastrocytomas in 40% of the mice, with 60% of *NES*-tva mice developing similar gliomas [10]. When these experiments were undertaken in *Cdkn2a*-null mice, the gliomas formed with a shorter latency and were of higher grade [20]. On the other hand, injection of adenovirus containing the *EGFR*vIII mutant into mice harboring activated RAS led to efficient formation of glioblastoma [21].

Lentiviruses expressing oncogenes such as *HRAS* or *AKT* were efficiently introduced into mice expressing Cre-recombinase under varying promoters such as *GFAP*. Glioblastoma tumors were efficiently formed when lentiviruses harboring activated *RAS* and *AKT* were injected into mice expressing *GFAP*-Cre on a *Trp53* heterozygous background [22, 23]. Current studies have allowed the determination that the gliomagenesis potential of mice is greater at a younger age with excision of glioblastoma-relevant genes such as *PTEN, NF1*, and *TP53* [24]. Misuraca et al. [25] have established low- and high-grade glioma, which phenocopies diffuse intrinsic pontine glioma, resulting from injection of Pax3-Tv-a;*Trp53* ^fl/fl^ mice with RCAS-*PDGFB* and RCAS-Cre, with or without RCAS-H3.3K27M. In the RCAS/Tv-a glioma mouse model, avian retroviruses (produced from RCAS plasmids) infect mouse cells expressing Tv-a (the receptor for RCAS viruses) [26].

### Xenograft and allograft models

For many years, immunodeficient rodents have been an important tool in modeling human glioblastoma. Propagation and testing of glioblastoma in such animals is most commonly accomplished in the subcutaneous flank location (heterotopic), although recent years have seen increased use of orthotopic (intracranial) xenograft models. For both heterotopic and orthotopic studies, xenograft and allograft tumors are usually established from permanent human glioblastoma cell lines.

Invasive orthotopic xenografts have also been established from surgical specimens that were first maintained as tissue spheroids in short-term culture [27, 28]. Finally invasive intracranial tumors have been established from heterotopic xenografts generated by direct transplantation of surgical specimens and subsequently sustained by serial passaging in the flanks of nude mice [29, 30].

Glioblastomas that have been continuously propagated as flank tumors recapitulate this very important and characteristic feature of human glioblastoma following intracranial transfer. Different from the heterotopic transplantation, the direct orthotopic transplantation denies the influences of in vitro culture, provides a proper microenvironment, and preserves the integrity of tumor-initiating cells [31, 32].

Many human and mouse cell lines have been used in xenograft and allograft models (Table 2). Tateishi et al. [33] used the SCID mouse to study the vulnerability of IDH1-mutant cancers to NAD + depletion. Ashizawa et al. [34] used NOD-SCID mice and NOG mice to study the effect of the STAT3 inhibitor STX-0119 on the proliferation of cancer stem-like cells derived from recurrent glioblastoma. Other mouse strains such as athymic nude (Nu/Nu) mice [35–37], CD1 nude mice [38], and athymic nude Foxn1-nu mice have also been used [39–41].

**Table 2:** Trends in xenograft and allograft mouse models of glioma

No	Study	Type	Cell line	Tumor histrogy	Genetic change	Animal model	Therapy	Drug administration method	Injection point of cells	Time of initiating the therapy	Duration of treatment	Observation period
1	Tateishi et al. [33]	Xenograft	MGG152(TIC*), HT1080(Human cell line)	Recurrent glioblastoma fibrosarcoma	IDH1 mutant(MGG152) IDH1 R132C mutant (HT1080)	SCID mice 7–10-week old female	NAMPT inhibitor	Oral administration (MGG152) intraperitoneal injection (HT1080)	Right striatum (MGG152) right flank (HT1080)	one week (MGG152) tumor diameters reached 5 mm (HT1080)	1 × /week (MGG152) 4 days/week (HT1080)	About 30 days (MGG152) 17 days (HT1080)
2	Ashizawa et al. [34]	Xenograft	GB-SCC010 GB-SCC026 (primary glioblastoma stem cell lines from patients)	Primary glioblastoma	NA*****	NOD-SCID** mice, NOG*** mice 5–6 week old	STAT3 inhibitor	Oral administration	Subcutaneous	Bearing tumor of > 35mm^3^	Daily/three weeks	28 days
3	Wykosky et al. [37]	Allograft	Primary ink4a/arf-/- astrocyte (mouse cell line)	Similar to glioblastoma	ΔEGFR-expressing and PTEN wild-type	Athymic mice 6-8 week old female	EGFR inhibitor(gefitinib)	Oral gavage	Cerebral (2 mm lateral and 1 mm anterior to the bregma)	20 days	5 days per week	About 50 days
4	Plowman et al. [35]	Xenograft	SF-295(Human cell line) U251(Human cell line)	Glioblastoma	NA*****	Athymic mice	Temozolomide BCNU	Oral gavage(TMZ) tail vein injection(BCNU)	Right cerebral hemisphere	1 day	day 1, day 5, day 9	90 days
5	Szabo et al. (2016) [39]	Xenograft	LNT-229 (Human cell line) LN-308(Human cell line)	Glioblastoma	Silencing microRNA-adapted shRNA	CD1-Foxn1^nu^ nude mice 6-12 week old	Neutralizing antibodies to VEGF or PIGF	Intraperitoneal injection	Right striatum	NA*****	Twice weekly	60 days
6	Sharpe et al. [41]	Xenograft	BT111(TIC*) BT116(TIC*)	Primary glioblastoma	Unmethylated MGMT(BT111) NA*****(BT116)	NU-Foxn1^nu^ nude mice	Monoamine oxidase B-activated pro-drug	Tail vein injection	Flank postglenoid foramen	4 weeks (flank model) 90 days (intracranial model)	day 0, day 12, day 23 (flank model) day 115, day 123, day 131 (intracranial model)	36 days (flank model) 307 days(intracranial model)
7	Zeng et al. [58]	Allograft	GL261-Luc(mouse cell line)	Glioblastoma	NA*****	C57BL/6J mice 6-8 week old	Radiation plus anti-PD-1 antibody	Intraperitoneal injection	left striatum(1 mm lateral and 1 mm anterior to the bregma, 3 mm deep from the cortical surface)	10 days	day 10, day 12, day 14	90 days
8	Zhang et al. [59]	Xenograft and allograft	LN-319(Human cell line) GL261(mouse cell line) [51]	Glioblastoma	NA***** (LN-319) ErbB2 expression(GL261)	NSG**** mice (LN-319) C57BL/6 mice(GL261)	ErbB2/HER2 -Specific NK Cells	Intratumoral injection	Subcutaneous right striatum (depth of 3 mm)	7 days	Weekly for 11 weeks (LN-319) weekly for 3 weeks(GL261)	303 days (LN-319) 200 days (GL261)
9	Parrish et al. [60]	Xenograft	GBM12(TIC*) [67]	Primary glioblastoma	MGMT hypermethylated	NA*****	PARP inhibitor (rucaparib) Temozolomide	Intraperitoneal injection oral gavage	Flank cerebral	NA*****	Days 1–5 every 28 days for 3 cycles	121 days (flank model) 81 days (intracranial model)
10	Gupta et al. [61]	Xenograft	GBM12(TIC*) [67]	Primary glioblastoma	TMZ-mgmt High TMZ-mgmt Low	Athymic mice	PARP inhibitor(veliparib) Temozolomide		Flank	Tumor of ~ 100 ± 15mm^3^	5 days every 28days for 3cycles	About 50 days
11	Garros-Regulez et al. [62]	Xenograft	U251(Human cell line)	Glioblastoma	NA*****	Foxn1^nu^-Foxn1^nu^ nude mice 8 week old	mTOR inhibition(rapamycin) Temozolomide	Intraperitoneal injection	Flank	1 week	Twice weekly for 12 weeks	About 60 days
12	Hashizume et al. [63]	Xenograft	SF7761(TIC*) SF8628(TIC*) GBM43(TIC*) [68]	Primary pediatric human glioma adult glioblastoma	H3F3A k27M mutation H3F3A k27M mutation MGMT unmethylated	Athymic mice 6 week old female	Demethylase inhibitor	Intraperitoneal injection	Flank brain stem	SF7761: about 50 days SF8628: 56 days GBM43: 5 days	SF7761: daily /10 days SF8628: daily /10 days GBM43: daily/7 days	SF7761: 160 days SF8628: 77 days GBM43: 18 days
13	Mathieu et al. [64]	Xenograft	Hs683 (Human cell line) U373 (Human cell line)	Glioma (Hs683) glioblastoma (U373)	NA*****	Nude mice (immunocompromised mice) 6 week old female	Bevacizumab Temozolomide	Tail vein injection oral administration	Cerebral	5 days	3 times per week for 3 consecutive weeks	80 days
14	Cho et al. [65]	Xenograft	LN443 (Human cell line)	Glioblastoma	Expressing EGFRvIII CT Del1 mutant (by retroviral infection)	SCID mice	Cetuximab erlotinib	Intraperitoneal injection oral administration	Right striatum	1 week	3 times per week	100 days
15	Yoshida et al. [66]	Xenograft	GBM39(TIC*) [69] U87(Human cell line) GBM12(TIC*) [69]	glioblastoma	EGFRvIII amplified (GBM39) expressing EGFRvIII (by retroviral infection:U87) wild-type EGFR(GBM12)	Athymic mice 5 week old female	Pan-ERBB inhibitor	Oral administration	Right caudate putamen	14 days (GBM39) 11 days (U87) 6 days (GBM12)	2 week (GBM39) 21 days (U87) day 10, day 13, day16,day 20, day 23 (GBM12)	66 days (GBM39) 32 days (U87) 70 days(GBM12)
16	Joo et al. [42]	Xenograft	Surgical specimens from glioblastoma patients	Glioblastoma	Depending on specimens	NOG*** mice	NA*****	NA*****	cerebral	With in 12 months	NA*****	About 200 weeks

*Patient-derived tumor initiating cell

**NOD/Shi-Parkdcscid

***NOD/Shi-scid IL-2Rγ-null

****NOD-SCID IL2Rγnull

*****not applicable

A library of orthotopic glioblastoma xenograft models using surgical samples of glioblastoma patients has been established. These patient-derived glioblastoma xenograft (PDX) tumors recapitulated histopathological properties and maintained genomic characteristics of parental glioblastoma in situ [42, 43]. Soeda et al. [44] reported a glioblastoma xenograft model containing heterogeneous subclones derived from a single tumor of a patient. This model may be useful for evaluating cell- and patient-specific drug responses. Patient-derived primary glioma cells might be a good solution but they are sometimes unable to maintain for long in culture and finding an accessible cell type for gliomas might be problematic.

Patient-derived stem cells are used to identify cell functions that are altered by disease, such as Alzheimer’s and Parkinson’s disease and thereby provide a target for drug discovery. Patient-derived glioblastoma stem cells have been generated from xenograft tumors of the glioblastoma surgically resected [45, 46]. Patient-derived glioblastoma stem cells are in nature, formed much larger neurospheres in a short period of time rather than patient-derived glioblastoma cells (not stem cells) [47]. Further, Sancho-Martinez et al. [48] have recently established human induced pluripotent stem (iPS) cells based glioma models in vivo.

Recent progress and expansion in next-generation sequencing (NGS) technologies enable to characterize the cancer genome in a time frame that is corresponding to treatment decisions, providing the chance to potentially increase the therapeutic effect by targeting the genomic alterations driving tumor behavior [49]. To challenge proposed therapy strategies on the patient of gliomas, the “Avatar mouse models”, which are based on the NGS data and generated as the individualized mouse xenografts by transplanting patient-derived tumor cells, have been investigated [50]. The development of PDX and patient-derived neurosphere/stem cell based xenograft models may approve bench testing of treatment strategies derived from the innovative genomic analysis.

There are important caveats to this approach that still need to be addressed in xenograft models. Firstly, the mice do not have an intact immune system. Inflammatory cells may be a critical component to the biology of the tumor and its response to certain drugs, particularly immunotherapy. Secondly, the surrounding stroma and microenvironment is of mouse origin, not human, and may interfere with drug response.

### Chemical carcinogen-induced models

Previously, only a chemical carcinogen-induced mouse model, the GL261 model, have been derived from an intracranially-induced methylcholanthrene tumor in C57BL/6 mice [51]. Recently, Johanns et al. [52] have reported that, by the assessment of the ability of the epitopes predicted in silico to be the highest affinity binders to activate tumor-infiltrating T cells harvested from GL261, they have found the mechanisms of the T cell–activating immune-directed therapy, presumably due to its hypermutator phenotype. The ability of gliomas to induce local and systemic immunosuppression restrict the innate defense against tumor growth and the efficacy of adaptive immunotherapy and thus presents a significant challenge to the development of innovative therapies [53].

### Spontaneous models

In 1971, H. Fraser has described the first incidence of a spontaneous glioma within the SMA-560 mouse strain. Initially, these tumors, which resembled human anaplastic astrocytomas, were restricted to in vivo studies only, as tumorigenicity was lost with repeated in vitro passaging of tumor explant cultures [54].

## Conclusions

Glioblastoma is one of the most problematic cancers to treat. Despite advances in molecular profiling of the disease, information is still lacking, particularly regarding treatment.

As genome-wide sequencing efforts continue in humans, mouse glioma models that better recapitulate the complex genomic landscape of human glioma will be generated. These models will provide increasingly powerful tools for the validation of hypotheses engendered by human genomic data, such as confirming the driver mutations that are causal to oncogenesis, as well as for preclinical testing of personalized therapy.
